# The transdifferentiation of human dedifferentiated fat cells into fibroblasts: An in vitro experimental pilot study

**DOI:** 10.1097/MD.0000000000037595

**Published:** 2024-03-29

**Authors:** Nam Kyu Lim, Hong Bae Jeon, Sungyeon Kim

**Affiliations:** a Department of Plastic and Reconstructive Surgery, Dankook University College of Medicine, Cheonan, Chungcheongnamdo, Republic of Korea; b Dankook Physician Scientist Research Center (DPSRC), Dankook University Hospital, Cheonan, Chungcheongnamdo, Republic of Korea.

**Keywords:** adipocytes, cell transdifferentiation, fibroblasts, skin

## Abstract

**Background:**

Skin grafting is a common method of treating damaged skin; however, surgical complications may arise in patients with poor health. Currently, no effective conservative treatment is available for extensive skin loss. Mature adipocytes, which constitute a substantial portion of adipose tissue, have recently emerged as a potential source of stemness. When de-lipidated, these cells exhibit fibroblast-like characteristics and the ability to redifferentiate, offering homogeneity and research utility as “dedifferentiated fat cells.”

**Methods and results:**

We conducted an in vitro study to induce fibroblast-like traits in the adipose tissue by transdifferentiating mature adipocytes for skin regeneration. Human subcutaneous fat tissues were isolated and purified from mature adipocytes that underwent a transformation process over 14 days of cultivation. Microscopic analysis revealed lipid degradation over time, ultimately transforming cells into fibroblast-like forms. Flow cytometry was used to verify their characteristics, highlighting markers such as CD90 and CD105 (mesenchymal stem cell markers) and CD56 and CD106 (for detecting fibroblast characteristics). Administering dedifferentiated fat cells with transforming growth factor-β at the identified optimal differentiation concentration of 5 ng/mL for a span of 14 days led to heightened expression of alpha smooth muscle actin and fibronectin, as evidenced by RNA and protein analysis. Meanwhile, functional validation through cell sorting demonstrated limited fibroblast marker expression in both treated and untreated cells after transdifferentiation by transforming growth factor-β.

**Conclusion:**

Although challenges remain in achieving more effective transformation and definitive fibroblast differentiation, our trial could pave the way for a novel skin regeneration treatment strategy.

## 1. Introduction

Clinically, skin grafting is the most common method for reconstructing damaged skin; however, if the overall health condition is poor, there is a significant risk of surgical complications.^[1]^ While other approaches involving skin substitutes or growth factors exist, their regenerative capacity is limited in cases of extensive damage, often necessitating additional surgeries, such as skin grafting.^[2–4]^ Some products cultivated from fibroblasts are promising but face challenges when applied to extensive injuries.^[5,6]^ Without proper support from the extracellular matrix and scaffold during transplantation, these materials can trigger inflammation, leading to a sharp decline in engraftment rates and raising doubts about their effectiveness.^[3,7]^ Currently, there is no established method for effective conservative treatment of extensive skin loss. Anatomically, a layer of adipose tissue exists beneath the skin.^[8]^ The surge in today’s overweight population has introduced a new challenge in addressing surplus fat. What if there were a way to efficiently utilize this excess fat?

Successful skin regeneration involves a composite structure of cells (fibroblasts), a scaffold, and an extracellular matrix, with cell-based therapies as the foundation.^[7,9,10]^ Clinically, damaged skin must be removed to aid recovery, exposing the underlying fat layer.^[7,8,11]^ The utilization of exposed adipocytes for skin regeneration is promising, as adipose tissue can function as a scaffold and extracellular matrix for regeneration.^[12,13]^ Adipose tissue contains perivascular stem cells within the stromal compartment, and research over the last 2 decades has extensively explored its regenerative potential.^[14]^ However, these stem cells are heterogeneous due to their mixed location within the stromal compartment, which exists in small quantities and limits their yield.^[15,16]^ Recently, mature adipocytes, comprising approximately 40% of adipose tissue, have emerged as a potential source. After removing lipids from these cells, they adopt a fibroblast-like form and exhibit the ability to redifferentiate, offering relative homogeneity and high utility in research. Yagi et al^[17]^ introduced a preadipocyte cell line derived from fully developed adipocytes of mice. These cells were labeled as dedifferentiated fat (DFAT) cells. Subsequently, comprehensive research was conducted on DFAT cells to highlight their remarkable stemness. Similar to bone marrow-derived cells (BMDCs), DFAT cells have been demonstrated to possess the capacity for differentiating into diverse mesenchymal tissue lineages.^[15,16,18–21]^ Taking inspiration from this context, we conducted an in vitro study to explore the feasibility of using mature adipocytes to induce fibroblast-like characteristics in the adipose tissue and transdifferentiate them into skin tissue for skin regeneration in patients with skin defects.

## 2. Materials and methods

### 2.1. Preparation of DFAT cells using human adipocytes

Human subcutaneous fat tissues (10–30 g) were obtained from tissues discarded during flap surgery in accordance with the guidelines of the Institutional Review Board of Dankook University Hospital (IRB No. DKUH-2020-05-001). Between May 20, 2020, and May 19, 2021, except for 5 patients in whom fat collection was not possible, 8 of the 13 patients (mean age 36.25 ± 15.95 years, range 17–59 years) who provided consent prior to surgery were included in this study. All patients provided written informed consent, signed by their guardians if they were minors. None of the patients had hematologic viral infections, such as hepatitis B or C, syphilis, or human immunodeficiency virus. After rapid washing with Dulbecco’s modified Eagle’s medium (DMEM; Thermo Fisher Scientific, Inc., Waltham, MA), the adipose tissue was chopped into small pieces and incubated with 1 mg/mL type I collagenase (Thermo Fisher Scientific, Inc.) at 37°C for 60 minutes. After collagenase digestion, the desolate tissues were filtered through a 100 μm nylon sieve to get isolated mature adipocytes free of stromal-vascular components. The filtered cells were washed 3 or 4 times with DMEM and centrifuged for 5 minutes at 1350 rpm. After centrifugation and removal of the top oil layer, pure adipocytes were isolated from the floating second layer on the top. Then, these adipocytes were placed in a 25 cm^2^ cell culture flask that was entirely filled with DMEM containing both 20% fetal bovine serum (Thermo Fisher Scientific, Inc.) and 1% penicillin (Thermo Fisher Scientific, Inc.). Mature adipocytes attached weakly to the ceiling surface during the first 3 to 4 days at 37°C in a 5% carbon dioxide incubator. The cells began to lose their spherical form at approximately day 7. The medium was then replaced with fresh medium, and the flask was reinverted. The dedifferentiated fat cells were cultured for 14 days. During cultivation, a microscope and Oil Red O staining (Sigma-Aldrich, St. Louis, MO) were used to observe the process of cell differentiation.

### 2.2. Immunophenotype analysis with fluorescence-activated cell sorting (FACS) in DFAT cells

Functional verification of dedifferentiated adipocytes was performed using flow cytometry. Fluorescein isothiocyanate, phycoerythrin, or allophycocyanin-conjugated antibodies against CD34 (hematopoietic marker), CD36 (adipocyte progenitor marker), CD90 and CD105 (mesenchymal stem cell markers), and CD56 and CD106 (fibroblast markers) were used to stain DFAT cells harvested at day 14 (Passage 2, P2) (all markers were procured from BD Biosciences, San Jose, CA). Cells were examined using a CytoFLEX flow cytometer and the CytExpert software package (Beckman Coulter Life Sciences, Irvine, CA). Additionally, FACS was used to examine the function of DFAT cells after 14 days of transdifferentiation. The transdifferentiation procedure was as follows.

### 2.3. Induction of transdifferentiation in DFAT cells by TGF-β

P2 DFAT cells were seeded into 12-well plates (3 × 10^4^ cells per well) containing medium and exposed recombinant human transforming growth factor beta 1 (TGF-β; Bio-Techne, Minneapolis, MN) for differentiation trials. Cell cytotoxicity testing was performed prior to induction of DFAT cells using a Cell Counting Kit-8 assay. The cells were incubated for 24, 48, and 72 hours. Thereafter, 10 μL of Cell Counting Kit-8 solution was applied to the cells, followed by incubation at 37°C for 1 hour. A microplate luminometer was used to measure optical density at 450 nm. DFAT cells were cultured for 14 days at validated concentrations.

### 2.4. Functional verification of transdifferentiated DFAT cells using quantitative real-time polymerase chain reaction and western blot

After 14 days of DFAT cell transdifferentiation, total RNA was extracted using the RNeasy Mini Kit (QIAGEN, Venlo, Netherlands) according to the manufacturer’s instructions. Then, 1 μg of total RNA was reverse-transcribed into complementary deoxyribonucleic acid using the HyperScript™ II Reverse Transcriptase Master Mix (Thermo Fisher Scientific, Inc.), and the resulting complementary deoxyribonucleic acid was amplified by PCR as per the manufacturer’s instructions. All primers used for the mRNA analysis were designed using the National Center for Biotechnology Information nucleotide library (Table 1). We validated the fundamental components of fibroblast-secreted proteins including alpha-smooth muscle actin (α-SMA) and fibronectin at the RNA level. Glyceraldehyde 3-phosphate dehydrogenase was used as a stable housekeeping gene. Expression levels were quantified in terms of each other using the comparative C_t_ method.

**Table 1:** Human gene primers used in quantitative real-time polymerase chain reaction.

Gene	Forward primers	Reverse primers
α-SMA	CTTTCTACAATGAGCTTCGTG	ATTTGAGTCATTTTCTCCCG
Fibronectin	GTCAGCCCAACTCCCACC	TTGGTGGCCGTACTGCTG
GAPDH	CAATGCCTCCTGCACCACCA	GATGTTCTGGAGAGCCCCGC

Sequence: 5′ to 3′.

α-SMA = alpha-smooth muscle actin, GAPDH = glyceraldehyde 3-phosphate dehydrogenase.

Scraping grown cells from cell culture substrates in standard sample buffer enabled the isolation of proteins. Electrophoretic separation of the protein samples was performed on polyvinylidene difluoride membranes using SDS-PAGE (Thermo Fisher Scientific, Inc.). We used the primary antibodies for α-SMA, fibronectin, and vimentin provided by Abicam Corporation (Cambridge, UK). The results were exported to Prism version 8.0 (Graphpad, La Jolla, CA) for analysis.

### 2.5. Confocal fluorescence microscopy

Cell samples were fixed in 3% paraformaldehyde for 10 minutes, washed with PBS, and then permeabilized with 0.2% Triton X-100 for 30 minutes for immunofluorescence. Primary antibodies directed against-SMA (mouse monoclonal antibody; Abicam Company, Cambridge, UK) were administered for 1 hour in 0.02% Triton X-100 buffer, followed by washing thrice. Goat antimouse IgG Alexa Fluor 568 (Thermo Fisher Scientific, Inc.) was used as the secondary antibody. A Fluoview FV 3000 (Olympus, Tokyo, Japan) was used to capture confocal images of the immunofluorescence-stained slices.

## 3. Results

### 3.1. Microscopic morphology and functional verification of DFAT cells

**Figure 1.**
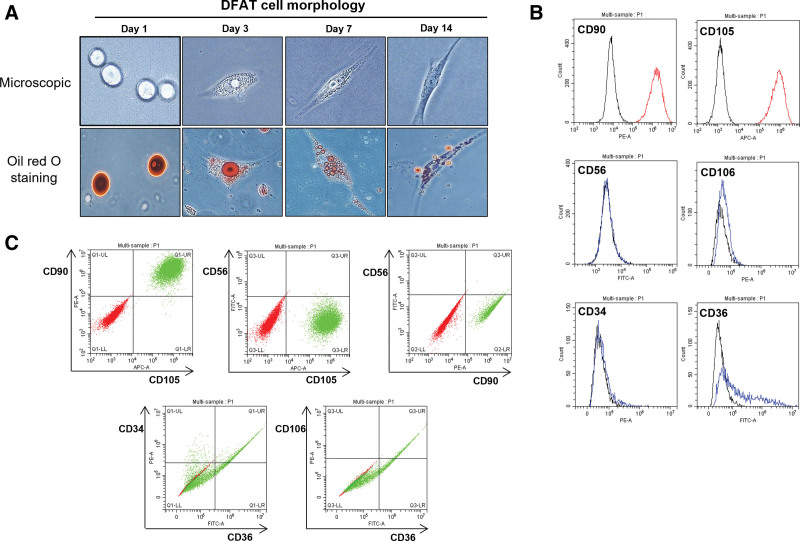
Microscopic morphology and functional verification of DFAT cells. (A) Microscopic morphology of DFAT cells. The lipid-laden material observed on the first day gradually began to degrade on the third and seventh days, and it was completely expelled from the cells on the 14th day, and transformed into fibroblast-like cells. Staining with Oil Red O provides a better perspective on this phenomenon. (B) Functional verification of DFAT cells using fluorescence-activated cell sorting on day 14. Over 95% of the cells were positive for CD90 and CD105, which are markers of cells in the mesenchymal stem cell. However, negative results were observed for CD56, CD106 (fibroblast markers), and CD34 (hematopoietic marker). More than 50% of CD36 (an adipocyte progenitor marker) were also negative. (C) Multifactor analysis of fluorescence-activated cell sorting. Red dots represent the control, whereas green dots represent DFAT cell expression. DFAT = dedifferentiated fat.

The microscopic appearance of DFAT cells at different time points revealed gradual degradation of lipid-laden material, which commenced on day 1, progressed through day 3 and 7, and culminated in complete expulsion by day 14. Consequently, transformation into fibroblast-like cells was observed. Enhanced visualization of this process was achieved using Oil Red O staining (Fig. 1A). Functional validation of DFAT cells was conducted on day 14 using FACS. Remarkably, ≥95% of cells exhibited positive expression for CD90 and CD105, both recognized as markers of mesenchymal stem cells (MSCs). Conversely, negative outcomes were noted for CD56 and CD106 (fibroblast markers), and CD34 (hematopoietic marker). Additionally, over 50% of CD36 (an adipocyte progenitor marker) displayed negative expression (Fig. 1B). The multifactor FACS analysis is shown in Figure 1C.

### 3.2. TGF-β induced transdifferentiated DFAT cells

**Figure 2.**
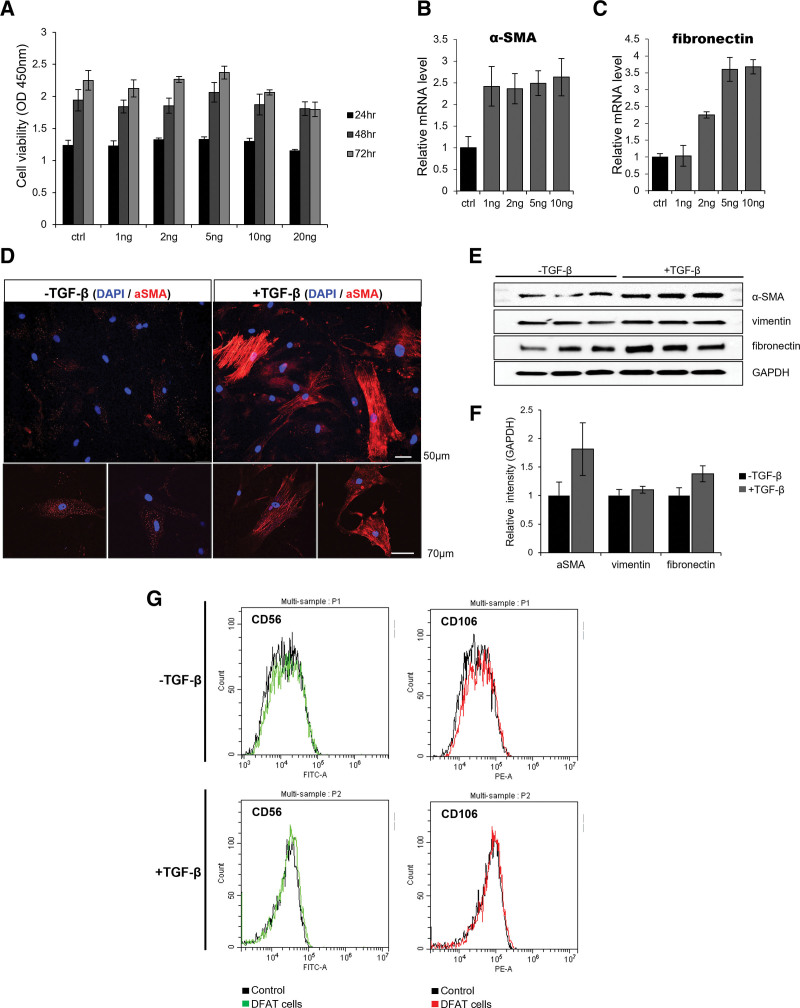
Induction of transdifferentiation in DFAT cells by TGF-β and comparative analysis of its efficacy. (A) DFAT cells on day 14 served as controls, and various concentrations of TGF-β (1, 2, 5, 10, and 20 ng) were used to indicate cell viability (absorbance measured at 450 nm using a microplate reader). The maximum cell viability was observed at 5 ng, with decreasing viability at 10 and 20 ng. (B) Relative mRNA level of α-SMA by RT-PCR. Compared with the control group, the increase from 1 to 10 ng was more than twice as high. (C) Relative mRNA levels of fibronectin determined by RT-PCR. In contrast to α-SMA, it continued to rise to 5 ng and then steadied at 10 ng. (D) Comparison of TGF-β treated and untreated DFAT cells using confocal fluorescence microscopy. Transdifferentiation was induced with 5 ng TGF-β during a 2-week period, and α-SMA expression was enhanced in comparison to DFAT cells not treated with TGF-β. (E and F) Protein analysis by western blot between TGF-β treated and untreated DFAT cells. While α-SMA and fibronectin were highly expressed in DFAT cells treated with TGF-β, vimentin was not substantially changed. (G) Fluorescence-activated cell sorting analysis of the functional verification of DFAT cells with or without TGF-β treatment. Both cells represented negative result for CD56 and CD106 (fibroblast marker). α-SMA = smooth muscle actin, DFAT = dedifferentiated fat, TGF = transforming growth factor.

On day 14, DFAT cells were designated as controls, and various concentrations of TGF-β (ranging from 1 to 20 ng) were employed to evaluate cell viability, measured at 450 nm through a microplate reader. Notably, the peak cell viability was observed at 5 ng/mL, with viability declining at 10 and 20 ng/mL (Fig. 2A). Shifting the focus to gene expression, relative mRNA levels of α-SMA were assessed using RT-PCR, uncovering a more than twofold increase from 1 ng to 10 ng/mL compared to the control group (Fig. 2B). In contrast, fibronectin mRNA levels increased up to 5 ng/mL and stabilized at 10 ng (Fig. 2C). Consequently, we established 5 ng/mL as the optimal concentration for in vitro differentiation and induced the differentiation of DFAT cells for subsequent experiments. The influence of TGF-β on transdifferentiation was explored using confocal fluorescence microscopy, revealing heightened α-SMA expression in DFAT cells treated with 5 ng/mL TGF-β over a 2-week period. Subsequent protein analysis via western blot demonstrated elevated α-SMA and fibronectin expression in TGF-β-treated DFAT cells, while vimentin displayed minimal alterations (Fig. 2D–F). However, in contrast to the observed microscopic disparities, the functional validation of DFAT cells, conducted using FACS, yielded negative outcomes for the fibroblast markers CD56 and CD106 in both TGF-β-treated and untreated cells (Fig. 2G).

## 4. Discussion

Fibroblasts play a crucial role as essential interstitial cells, and are involved in a diverse array of biological functions. Its responsibilities encompass providing essential structural support to tissues, secreting the extracellular matrix, contributing to the mending of tissue damage, and actively participating in immune responses.^[22,23]^ The intricate process of skin regeneration involves a multifaceted interplay between cells, scaffold structures, and the extracellular matrix, in which fibroblasts play an indispensable role.^[6,7,9,10,24]^ Similar to other organs such as the heart, lungs, gastrointestinal tract, muscles, and adipose tissues, the skin emerges from mesenchymal-derived connective tissues.^[23]^ Because of this fundamental relationship, numerous efforts have been undertaken to reprogram fibroblasts using strategies involving the utilization of induced pluripotent stem cells.^[25–27]^

When devising an optimal in vivo reprogramming strategy, it is beneficial to establish key milestones against which the actual outcomes can be measured. The initial milestone involves identifying the most suitable cell type source capable of transforming into the desired cell type, strategically positioned within the appropriate locale. The second milestone encompasses the identification of the most effective reprogramming pathway, necessitating the identification of a minimal set of reprogramming factors that are essential for establishing the desired cell-type program. The third milestone entails generating cells that mirror the molecular identity and phenotype of the desired cell type while erasing their original cellular characteristics. Partial phenotypic expression might fall short of rectifying organ function deficits and could potentially yield negative repercussions. Given the variations in host environments and their potential hindrances to seamless integration, the fourth milestone centers on recognizing potential environmental constraints. Finally, the fifth milestone involves the successful functional integration of reprogrammed cells, thereby reinstating lost organ functions.^[25,28]^

In the context of the aforementioned first milestone, a range of cell types with the potential for tissue regeneration have been identified, underscoring the importance of judicious cell source selection for clinical applications. MSCs are versatile somatic stem cells that are capable of differentiating into osteoblasts, chondrocytes, adipocytes, and myocytes.^[29]^ Adipose-derived stem cells (ASCs), which exhibit multilineage potential comparable to that of BMDCs, offer the advantage of easy accessibility to mature adipose tissue.^[15,16]^ Nonetheless, ASCs are heterogeneous and stem from the nonadipocyte fraction within the adipose tissue, including the stromal-vascular fraction. Moreover, early passaged ASCs contain contaminating endothelial cells, smooth muscle cells, and pericytes. High-purity cell sources are imperative for harnessing these stem cells for widespread clinical use.^[15,16,20,30]^ Mature adipocytes constitute the majority of adipose tissue cells and are typically considered stable, having reached terminal differentiation.^[20]^ However, Yagi et al^[17]^ established a preadipocyte cell line, termed DFAT cells, derived from mature adipocytes of mice, demonstrating that these cells possess vigorous proliferative activity and have the potential to differentiate into various mesenchymal tissue lineages, such as BMDCs. Notably, other studies have highlighted that DFAT cells exhibit superior MSC-like properties compared with ASCs from the same adipose tissue source. Although DFAT cells showed over 99.5% homology with ASCs in comprehensive gene expression analysis and shared a similar humoral factor secretion profile, they still exhibited elevated secretion levels of hepatocyte growth factor, vascular endothelial growth factor, stromal cell-derived factor 1, and leptin, correlating with a robust and consistent angiogenic effect. This unique property of DFAT cells stems from their isolation by ceiling culture, which exploits adipocyte buoyancy.^[15,16,21]^ Consequently, DFAT cells are considered to be a homogeneous cell population, underpinning their potential utility in various clinical applications.

Based on these insights, we aimed to reprogram DFAT cells into fibroblasts of the same differentiation lineage, recognizing their pivotal roles in skin regeneration. We embarked on this endeavor with a minimalistic approach, aiming to achieve the second milestone by utilizing the fewest possible reprogramming factors. Understanding the mechanisms governing adipogenic differentiation and its reversal is invaluable for identifying critical reprogramming factors. The process of adipocyte differentiation involves a precisely orchestrated sequence of temporal gene expression events. Notably, considerable attention over the last 2 decades has centered around the pivotal involvement of the nuclear receptor PPARγ and members of the C/EBP family in driving adipogenesis.^[31]^ However, it is crucial to underline that the efficient functionality of these C/EBP transcription factors hinges on the presence of PPARγ. Furthermore, TGF-β1, a notable player, has garnered recognition as a significant regulator of adipocyte physiology due to its role in impeding adipogenesis. Beyond its inhibitory role, this growth factor plays a pivotal role in the remodeling of adipose tissue by inducing the expression of genes encoding extracellular matrix proteins such as collagen.^[20]^ TGF-β orchestrates the differentiation pathways of numerous cell types, including adipocytes.^[32]^ Although the signaling pathways downstream of these factors share common properties, specific family members have divergent effects on cellular fate. TGF-β superfamily proteins bind to serine/threonine kinase receptors, engaging in both SMAD-dependent and SMAD-independent mechanisms. Phosphorylation of receptor-regulated SMADs, such as SMAD1 or SMAD3, triggers dimer formation with SMAD4, leading to nuclear translocation where these SMAD proteins govern target gene transcription. TGF-β and its signaling components are expressed in cultured adipocytes and adipose tissue. However, TGF-β inhibits the differentiation of preadipocytes in vitro, and transgenic overexpression of TGF-β hampers adipose tissue development.^[33]^ This inhibitory effect of TGF-β is executed through SMAD3 phosphorylation, while conversely, bone morphogenetic protein-2 promotes adipogenesis by inducing the nuclear localization of Schnurri-2, SMAD1, and C/EBPα.^[31]^ A discovery by Tchernof and coworkers^[20]^ revealed significantly higher TGF-β1 and collagen type I alpha 1 gene expressions in DFAT cells when compared to whole adipose tissue samples and stromal-vascular fraction cells. In a study utilizing the 6-well plate model, incubation of cells with TGF-β (5 ng/mL) during dedifferentiation notably elevated the gene expressions of TGF-β1, TGF-β2, collagen type I alpha 1, and COL6A3 compared to cells cultured with 5% serum alone (*P* < .05 for all). However, a definitive causal link between TGF-β/SMAD signaling, and dedifferentiation was not established in their study.^[20]^ Furthermore, we tried on the transdifferentiation of DFAT cells to fibroblast by applying TGF-β at a concentration of 5 ng/mL. Using a suspension culture approach, we induced differentiation and performed comprehensive functional assessments using specific cell markers.

Validating the phenotype and function of these cells aligns with the accomplishment of the third milestone. Under standard culture conditions, MSCs are expected to adhere to culture substrates. Phenotypically, they should express CD73 (ecto 5′-nucleotidase), CD90 (Thy-1), and CD105 (Endoglin), while concurrently demonstrating the absence of CD45 (protein tyrosine phosphatase), CD34 (hematopoietic cluster differentiation molecule 34), CD14 (macrophage/neutrophil cluster differentiation molecule 14), CD19 (follicular dendritic cell/B-cell cluster differentiation molecule 19), and human leukocyte antigen-DR.^[34]^ Although human skin fibroblasts exhibited a morphological profile comparable to that of DFAT cells and ASCs, the expression of CD105 and CD49d was attenuated. Significantly, fibroblasts demonstrated robust positivity for CD56 (neural cell adhesion molecule), distinguishing them from other cell types under scrutiny.^[35]^ The expression of CD106 (vascular cell adhesion molecule-1) is consistently absent in fibroblasts, ASCs, and DFAT cells.^[35,36]^ In our transdifferentiation experiments, CD90 and CD105 were used to validate stemness, whereas CD56 and CD106 were used to discern the transition to fibroblasts. Through comprehensive RNA and protein analysis, we observed an elevation in α-SMA and fibronectin expression in fibroblasts 2 weeks following differentiation induced by TGF-β in DFAT cells. However, we were unable to conclusively demonstrate that these cells directly differentiated into fibroblasts. However, these findings inevitably present limitations within the scope of this study. Our endeavors underscore the necessity of a more potent transformation process to effectively convert adipocytes into fibroblasts to overcome the third milestone. Furthermore, the assessment of the transformed cells relied solely on clusters of differentiation markers, prompting the need to validate these results through a broader array of analyses.

## 5. Conclusion

The objective of this study was to investigate the in vitro transdifferentiation of DFAT cells into fibroblasts. If we succeed in surmounting reprogramming challenges, we envision the potential for presenting this approach as a promising treatment avenue for skin regeneration.

## Author contributions

**Conceptualization:** Nam Kyu Lim, Hong Bae Jeon.

**Data curation:** Nam Kyu Lim, Hong Bae Jeon.

**Formal analysis:** Nam Kyu Lim.

**Funding acquisition:** Nam Kyu Lim.

**Investigation:** Nam Kyu Lim, Sungyeon Kim.

**Methodology:** Nam Kyu Lim, Hong Bae Jeon.

**Project administration:** Nam Kyu Lim.

**Resources:** Nam Kyu Lim.

**Software:** Nam Kyu Lim.

**Supervision:** Nam Kyu Lim.

**Validation:** Nam Kyu Lim, Hong Bae Jeon, Sungyeon Kim.

**Visualization:** Nam Kyu Lim.

**Writing – original draft:** Nam Kyu Lim.

**Writing – review & editing:** Nam Kyu Lim.
